# Supplementary material for: Mucin-Producing Lobular Breast Carcinoma Metastasis to an Ovarian Fibroma: Histopathological and Immunohistochemical Analysis of a Rare Case and Literature Review
Source: Diagnostics (Basel). 2024 Apr 30;14(9):953. doi: 10.3390/diagnostics14090953 (PMC11083407; doi:10.3390/diagnostics14090953)
Supplement: Supplementary file 1 [file diagnostics-14-00953-s001.zip › diagnostics-2950215-supplementary.pdf]

SPITALUL CLINIC MUNICIPAL DE URGENȚĂ TIMIȘOARA
ANMCS
unitate aflată în SUPRAVEGHEREA
Autorității Naționale de Management al Calității în Sănătate

SPITALUL CLINIC MUNICIPAL DE URGENȚĂ TIMIȘOARA
Număr Operator de date cu caracter personal - 37058
Str. Hector Nr. 2 A, Timişoara, Timiş - RO, 300041
Tel : 0256/200048, 0256/221553, Fax : 0256/200046
http://www.spitalul-municipal-timisoara.ro
E-mail: secretariat@smtm.ro Cod fiscal: 4483447

ISO 9001
ISO 14001
ISO 45001
IAS ACCREDITED

NR.E-628/31.01.2024

ACORDUL DOMNULUI CONF. DR. MALIȚA DANIEL CLAUDIU – MANAGERUL SPITALULUI CLINIC MUNICIPAL DE URGENȚĂ TIMIȘOARA,

Prin prezenta se confirmă acordul domnului MANAGER CONF. DR. MALIȚA DANIEL CLAUDIU privind desfasurarea protocolului de studiu: '' A rare case of metastasis from mucin producing lobular breast carcinoma to an ovarian fibroma:a case report and review of the literature''.-Serviciu Anatomie Patologică I

Investigatori: Conf.Univ.Dr.FAUR ALEXANDRA,MD ANATOMIE PATOLOGICĂ I
AVIZ: ŞEF SECŢIE CLINICĂ DE CHIRURGIE GENERALĂ II-ONCOLOGICĂ II Conf.Univ.Dr.ILINA RAZVAN
AVIZ: ŞEF SERV. INFORMATICĂ ING. MUNTEANU ADINA

Acordul unitatii pentru desfasurarea studiului clinic este favorabil cu conditia respectării legislației în vigoare, a ghidurilor de bună practică, a legii drepturilor pacientului, a confidențialității Regulament nr. 679 din 27 aprilie 2016 privind protecţia persoanelor fizice în ceea ce priveşte prelucrarea datelor cu caracter personal şi privind libera circulaţie a acestor date şi de abrogare a Directivei 95/46/CE (Regulamentul general privind protecţia datelor), a publicării rezultatelor.

MANAGER,

CONF. DR. MALIȚA DANIEL CLAUDIU

Direcția de Sănătate Publică a Jud. Timiș
Spitalul Clinic Municipal de Urgență Timișoara
1

SERV. JURIDIC ŞI CONTENCIOS ADMINISTRATIV

ŞEF SERV. C.J. FODOR ADELINE
